# Examining rare side effects in pooled trial data: symptomatic osteonecrosis in Hodgkin lymphoma

**DOI:** 10.18632/oncotarget.26486

**Published:** 2018-12-25

**Authors:** Sven Borchmann, Andreas Engert

**Affiliations:** Andreas Engert: Department of Internal Medicine and German Hodgkin Study Group, University Hospital of Cologne, Cologne, Germany

**Keywords:** Hodgkin lymphoma, lymphoma, osteonecrosis, avascular necrosis, survivorship

Hodgkin lymphoma has become a highly curable disease in a predominantly young patient population. Therefore, current clinical research in newly diagnosed patients aims to reduce treatment toxicity while maintaining a high cure rate [1]. This could be achieved by using new biological insights to develop novel treatments, attempting to improve risk stratification and treatment response monitoring, or by limiting an individual patient’s treatment exposure to the minimum necessary to achieve a cure [2-4].

Avascular osteonecrosis is a treatment complication that has not been thoroughly evaluated or recognized despite its potentially debilitating outcome. A recently published study of the German Hodgkin Study Group (GHSG) set out to provide a comprehensive evaluation of osteonecrosis in newly diagnosed Hodgkin lymphoma patients.

In our study [5], we evaluated the incidence, risk factors and timing of symptomatic osteonecrosis in Hodgkin lymphoma and described the location of its occurrence, interventions and outcome. Altogether, 11,330 patients who had been treated within the randomized GHSG phase III trials HD10-HD15 and HD18 were included. We identified 66 cases of osteonecrosis, of which 83.3% of cases occurred within 3 years of treatment commencement. There was a striking difference in the incidence between early-stage and advanced-stage Hodgkin lymphoma. The incidence of symptomatic osteonecrosis was 0.2% in early-stage but 1.0% in advanced-stage patients. We hypothesized that this difference might be caused by certain patient characteristics and the different treatment regimens used for early- and advanced-stage disease rather than the extent of disease per se.

We performed logistic regression analysis to identify patients who are at increased risk for osteonecrosis. The most important risk factor was the cumulative corticosteroid dose used, given either as part of the treatment or as a supportive measure (Odds ratio (OR): 1.31 (1.19-1.44) for each additional gram of prednisone received). Interestingly, we also observed a strong negative interaction between the cumulative corticosteroid dose with age (OR: 0.92 (0.86-0.99) for each additional 10 years of age). Most advanced-stage patients in our cohort were treated with variants of the BEACOPP (bleomycin, etoposide, doxorubicin, cyclophosphamide, vinblastine, prednisone, procarbazine) regimen, a highly effective treatment for Hodgkin lymphoma. This regimen contains prednisone for the first two weeks of every 3-week cycle, a factor that likely explains the observed difference in the incidence of osteonecrosis between early- and advanced-stage patients [6]. Male patients also had a higher risk for developing osteonecrosis compared to female patients (OR: 2.85 (1.10-7.40)). Other potential risk factors, such as obesity, the International Prognostic Score (IPS) and radiotherapy did not increase a patient’s risk for developing osteonecrosis.

The most commonly affected location was the femoral head and proximal femur (72.7%), followed by the knee including adjacent bones (17.5%). Other locations were rare. Over half of all patients with osteonecrosis (56.1%) needed some form of surgical intervention with most surgical interventions (84.4%) being either primary or, after failure of other measures, secondary joint replacements.

Whilst our study is the largest study of osteonecrosis as a treatment complication in Hodgkin lymphoma, it needs to be acknowledged that osteonecrosis was not a prespecified endpoint in the included studies and underreporting is not excluded in our cohort. It is for this reason that we only included symptomatic cases of osteonecrosis in our study, to mitigate the possible effect of underreporting. It is likely that if observational studies where designed with sensitive techniques, such as magnetic resonance imaging (MRI), and incorporated into specifically designed protocols that detection of non-symptomatic cases could in fact be much higher. Furthermore, the data on treatment of osteonecrosis and its outcome is based on a subset of patients for which data was available and thus might be subject to reporting bias also.

Osteonecrosis as a side-effect of the treatment of hematological malignancies has been comprehensively assessed in childhood acute lymphoblastic leukemia (ALL), which is usually treated with protocols containing relatively high doses of corticosteroids. Incidence rates have been reported to be much higher in childhood ALL than in our study [7, 8]. Interestingly, an important risk factor for developing osteonecrosis in childhood ALL is age, with teenagers being at higher risk than younger children [8]. Taken together with our findings, it seems that teenagers and young adults might be a high-risk group for osteonecrosis after corticosteroid containing cancer treatment. The incidence of osteonecrosis in Hodgkin lymphoma has been estimated to be higher in previous studies [9]. However, this was based on case series, at risk of having an overreporting bias. Interestingly, the largest previously reported cohort of Hodgkin lymphoma patients, in which the risk of osteonecrosis has been assessed, reported an incidence rate of 1.1%, similar to our study [10].

Our findings highlight the relevance of a thorough analysis of long-term side-effects of cancer treatment in the context of a disease that is highly curable. Long term follow-up of patients and collection of comprehensive data is necessary to identify previously under-recognized side-effects of treatments. This has implications for all stake-holders in clinical research. Firstly, regulators must carefully balance data protection with the need for long-term data collection and the accessibility of data for research groups. Secondly, pharmaceutical companies and academic research groups need to make their data more easily available (upon reasonable request) to researchers interested in studying specific side-effects or other questions across trial entities. Thirdly, research funders need to provide financial support for long-term patient follow up, with maintenance of data collection and data management for the clinical trials that they fund.

In Hodgkin lymphoma, individualized treatment by re-assessing a patient’s interim response during therapy has been successful in reducing treatment exposure for individual patients. For example, we have recently published results of the HD18 trial showing that the majority of advanced-stage HL patients can be treated with only 4 cycles of BEACOPP chemotherapy [6]. This alone has reduced the incidence of symptomatic osteonecrosis from 1.0% (6-8 cycles of BEACOPPescalated, 15/1455 patients) to 0.6% (4 cycles of BEACOPPescalated, 3/503 patients). We also recently published results of a phase 2 trial introducing the novel BrECADD regimen (brentuximab vedotin, etoposide, cyclophosphamide, doxorubicin, dexamethasone and dacarbazine) which substitutes short-term dexamethasone for long-term prednisone and may reduce the incidence of osteonecrosis further [11].

In our study we show that secondary examinations of pooled trial data can enable researchers to comprehensively analyze even rare side effects. This is reliant firstly on initial data collection being comprehensive enough, secondly, funding of suitable long-term follow up, material and data collection within the regulatory framework of the trial and, thirdly, collection of data on rare events from multiple trials by research-supportive data sharing practices.

In summary, we believe that our study is useful beyond Hodgkin lymphoma and provides clinicians with data that is helpful to identify cancer patients at higher risk for osteonecrosis after corticosteroid containing treatments.
